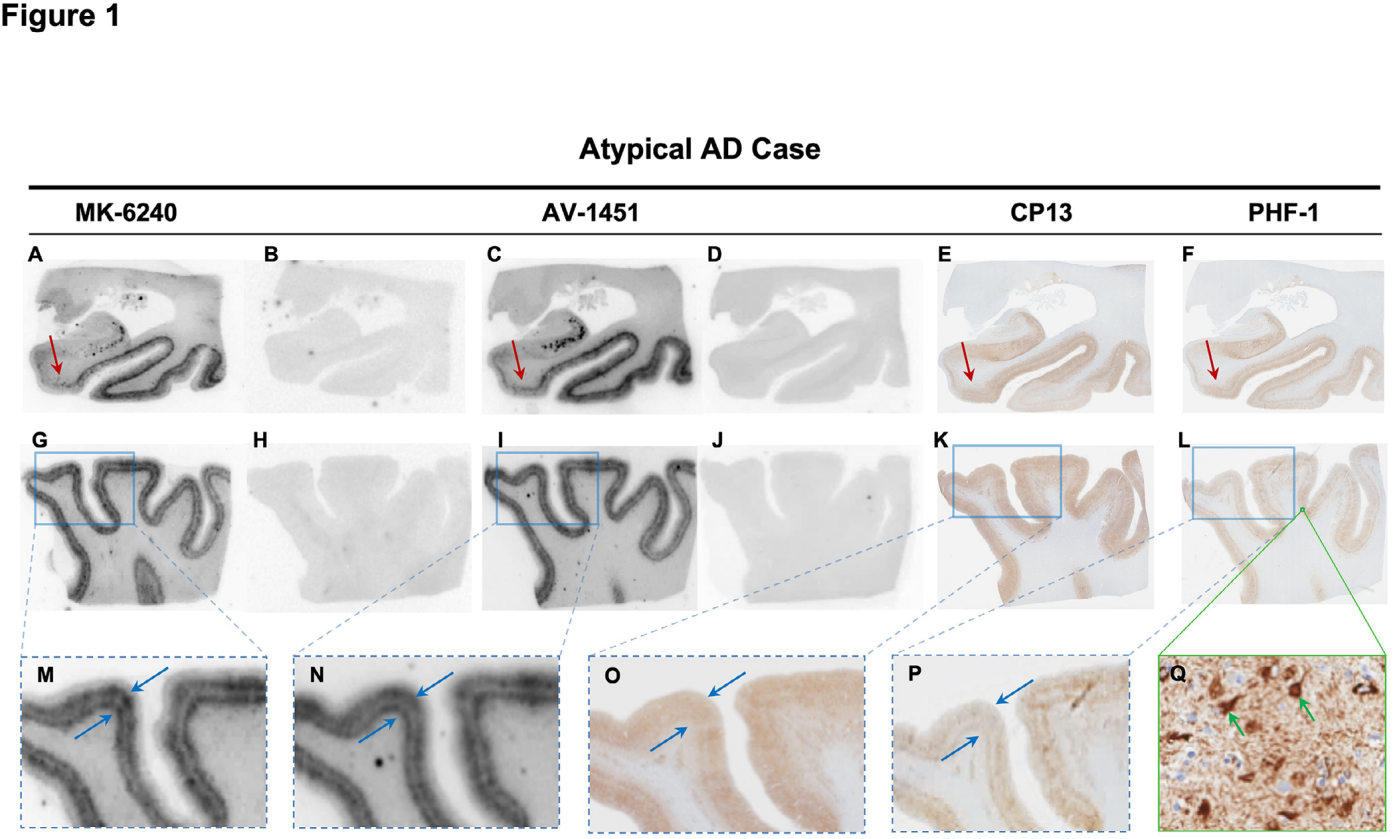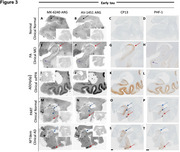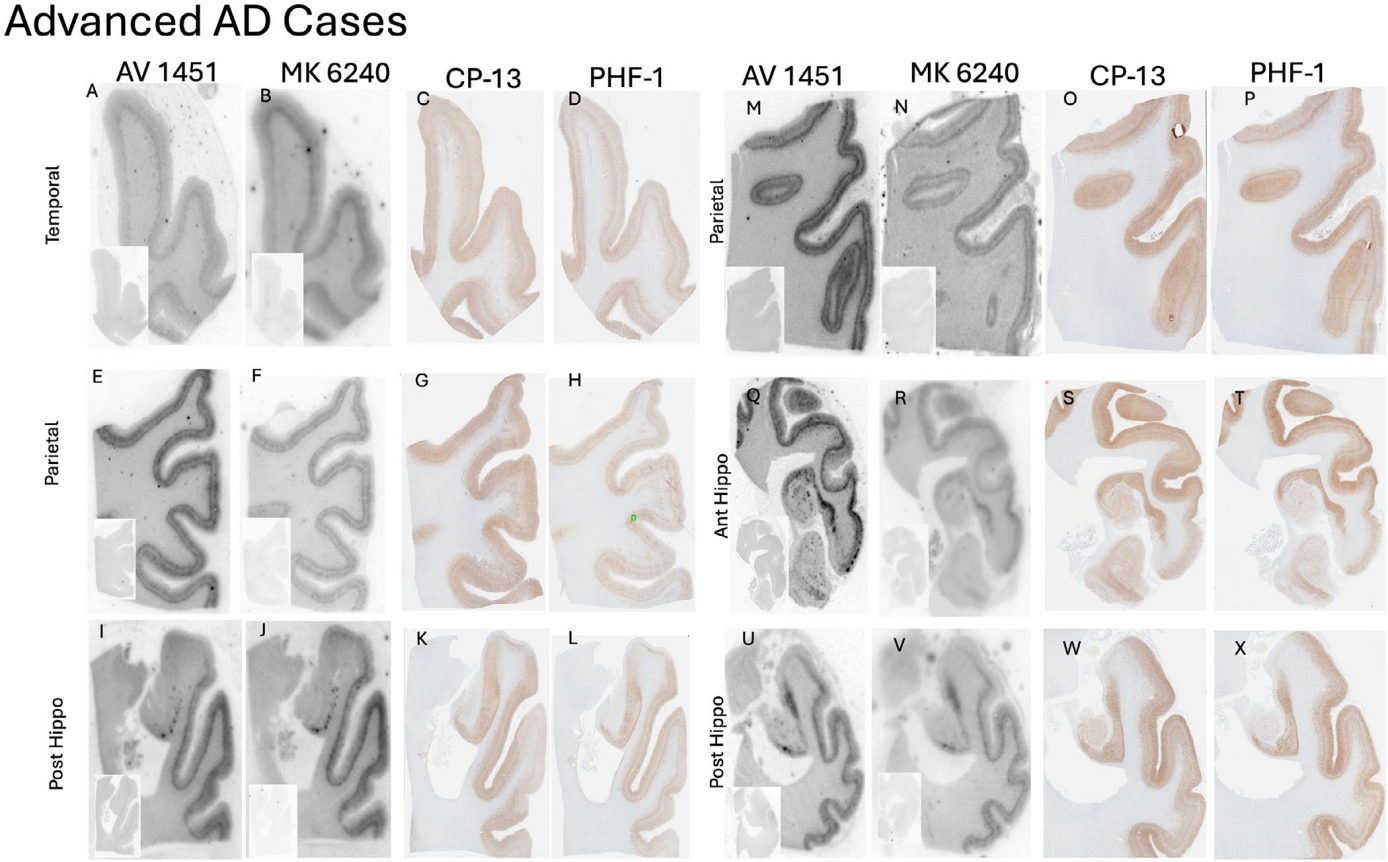# The Binding Patterns of Neurofibrillary Tangles Observed With High‐Resolution Autoradiography of ^18^F MK 6240 and ^18^F AV1451 Are Comparable and Predominantly Recognize Tangles at the Middle Stage

**DOI:** 10.1002/alz70856_106581

**Published:** 2026-01-10

**Authors:** Sujala Ghatamaneni, Courtney Hruska, Ian Shin, Tyler Bruinsma, Jeyeon Lee, Hoon‐Ki Min, Ping Fang, Christina M. Moloney, Ashley C. Wood, Eleni Constantopoulos, R. Ross Reichard, David T. Jones, Christopher G Schwarz, Jonathan Graff Radford, David S. Knopman, Clifford R. Jack, Ronald Petersen, Dennis W. Dickson, Melissa E. Murray, Val J Lowe

**Affiliations:** ^1^ Mayo Clinic, Rochester, MN, USA; ^2^ Mayo clinc, Rochester, MN, USA; ^3^ Mayo clinic, Rochester, MN, USA; ^4^ Hanyang Universtiy, Seoul, Gyeonggi, Korea, Republic of (South); ^5^ Department of Radiology, Mayo Clinic, Rochester, MN, USA; ^6^ Mayo Clinic, Jacksonville, FL, USA; ^7^ Department of Neurology, Mayo Clinic, Rochester, MN, USA

## Abstract

**Background:**

An intense search is underway for tau PET tracers with enhanced capability to visualize tau aggregates. Research indicates that [18F] AV1451 and [18F]MK 6240 exhibit comparable binding properties in terms of spatial distribution and severity of tau aggregates, as observed both in‐vivo and in‐vitro. This study focused on head‐to‐head pathological comparison of both the tracers binding specificity to tangle maturity levels (pretangles, mature tangles and ghost tangles) of tau aggregates as seen on autoradiography as compared to CP‐13 (early tangle maturity marker) and PHF‐1(middling tangle maturity marker) immunohistochemical (IHC) stains in Alzheimer's disease (AD) and non‐AD tauopathies.

**Method:**

Study participants included included those with AD and non‐AD tauopathies. Analyses were performed on serial 5um formalin fixed paraffin embedded postmortem human brain sections acquired from Mayo clinic brain bank. Visual assessment of colocalization with IHC as well as quantitative analyses were used. IHC sections were incubated with antibodies CP‐13 (recognizing early tangle maturity) and PHF‐1 (recognizing middling tangle maturity).

**Result:**

A total of 38 participants were examined. The participants included a spectrum from normal to AD, including mild cognitive impairment (MCI) and atypical AD. Participants with N279K (4R), P301L (4R), and R406W *MAPT* mutations (3R+4R) were included as well. Both the tracers showed strong binding to AD tau aggregates and no or minimal binding to non‐AD tauopathies. Generally, early tangle maturity tau accumulation participants had more tau demonstrated with CP‐13 than PHF‐1 and both the tracers [18F]MK 6240 and [18F]AV 1451 binding are well correlated with the PHF‐1 staining suggesting the preference for middling tangle level and minimal to early tangles.

**Conclusion:**

The emergence and validation of these new tau PET tracers have opened new opportunities for the development of more accurate diagnostic and management tools for AD. In our study on post‐mortem sections, [^18^F]MK‐6240 and [^18^F]Flortaucipir showed similar binding profiles with preference to middling tangle maturity levels.